# A tumor suppressor role for EZH2 in diffuse midline glioma pathogenesis

**DOI:** 10.1186/s40478-022-01336-5

**Published:** 2022-04-08

**Authors:** Swati Dhar, Samantha Gadd, Priyam Patel, Jake Vaynshteyn, G. Praveen Raju, Rintaro Hashizume, Daniel J. Brat, Oren J. Becher

**Affiliations:** 1 Department of Pediatrics, Simpson Querrey Biomedical Center, Stanley Manne Children’s Research Institute, Ann & Robert H. Lurie Children’s Hospital, Chicago, IL USA; 2 Department of Pathology, Simpson Querrey Biomedical Center, Stanley Manne Children’s Research Institute, Ann & Robert H. Lurie Children’s Hospital, Chicago, IL USA; 3 Quantitative Data Science Core, Center for Genetic Medicine, Northwestern University Feinberg School of Medicine, Chicago, IL USA; 4 Department of Pathology, Northwestern University Feinberg School of Medicine, Chicago, IL USA; 5 Department of Pediatrics and Department of Biochemistry and Molecular Biology, Simpson Querrey Biomedical Center, Stanley Manne Children’s Research Institute, Ann & Robert H. Lurie Children’s Hospital, Chicago, Northwestern University Feinberg School of Medicine, Chicago, IL USA; 6 Department of Pediatrics, Icahn School of Medicine at Mount Sinai, New York, NY USA; 7 Department of Neurology, Icahn School of Medicine at Mount Sinai, New York, NY USA; 8 Department of Oncological Sciences, Icahn School of Medicine at Mount Sinai, New York, NY USA; 9 Present Address: NeoImmuneTech Inc., 2400 Research Blvd., Suite 250, Rockville, MD USA

**Keywords:** Ezh2 loss-of-function, Ezh2 gain-of-function, Diffuse midline gliomas (DMGs), Tumor suppressor, Interferon gamma, Oxidative phosphorylation

## Abstract

Pediatric high-grade gliomas, specifically diffuse midline gliomas, account for only 20% of clinical cases but are 100% fatal. A majority of the DMG cases are characterized by the signature K27M mutation in histone H3. The H3K27M mutation opposes the function of enhancer of zeste homolog 2 (EZH2), the methyltransferase enzyme of the polycomb repressor complex 2. However, the role of EZH2 in DMG pathogenesis is unclear. In this study, we demonstrate a tumor suppressor function for EZH2 using *Ezh2* loss- and gain-of-function studies in H3WT DMG mouse models. Genetic ablation of *Ezh2* increased cell proliferation and tumor grade while expression of an *Ezh2* gain-of-function mutation significantly reduced tumor incidence and increased tumor latency. Transcriptomic analysis revealed that *Ezh2* deletion upregulates an inflammatory response with upregulation of immunoproteasome genes such as *Psmb8*, *Psmb9*, and *Psmb10*. *Ezh2* gain-of-function resulted in enrichment of the oxidative phosphorylation/mitochondrial metabolic pathway namely the isocitrate dehydrogenase *Idh1/2/3* genes. Pharmacological inhibition of EZH2 augmented neural progenitor cell proliferation, supporting the tumor suppressive role of EZH2. In vivo 7-day treatment of H3K27M DMG tumor bearing mice with an EZH2 inhibitor, Tazemetostat, did not alter proliferation or significantly impact survival. Together our results suggest that EZH2 has a tumor suppressor function in DMG and warrants caution in clinical translation of EZH2 inhibitors to treat patients with DMG.

**Supplementary Information:**

The online version contains supplementary material available at 10.1186/s40478-022-01336-5.

## Introduction

Deregulation of epigenetic modifier genes, such as enhancer of zeste homolog 2 (EZH2) has been causally linked to several cancers [16]. EZH2 is the methyltransferase component of the chromatin modifier polycomb repressor complex 2 (PRC2). EZH2 methylates lysine 27 of the H3 histone, which results in transcriptional silencing [13]. Although loss- and gain-of-function *EZH2* mutations have been documented in lymphomas, myeloid malignancies, leukemias, and solid tumors, only truncating mutations in the H3K36 methyltransferase *SETD2* [17] have been reported in pediatric high-grade gliomas (pHGG). Within this group, DMG, which constitutes approximately 20% of clinical pHGG, does not have any documented evidence of *EZH2* mutations [29]. Seminal large-scale genomic studies in DMG patients have established that somatic mutations in H3 histones, namely H3K27M (lysine 27 to methionine), serve as major drivers of the disease [5, 38, 47]. H3K27M histones can bind to the SET domain of EZH2 in vitro and induce global reduction of H3 di- and tri-methylation repressive marks on the chromatin [27]. However, focal gains in trimethylation marks have been associated with residual EZH2 activity [32]. Further, gain of H3K27M-K27Ac in actively transcribed gene loci excludes PRC2, challenging the notion that H3K27M can sequester PRC2 [39]. Therefore, the role of EZH2 in DMG remains unclear.

Functional outcomes from mutations/alterations in *EZH2* appear to be context dependent. A plethora of studies show the oncogenic functions of EZH2 in leukemias, lymphomas, and solid tumors ([23, 25, 26, 48]. On the other hand, EZH2 or SUZ12 depletion cooperates with other oncogenes to accelerate myelodysplastic syndrome and Ras-driven transcription in peripheral nerve sheath tumors hinting at a tumor suppressive function of the PRC2 complex [10]. Even in glioblastomas where EZH2 is overexpressed, prolonged depletion of *Ezh2* results in a switch in cell fate that promotes tumor progression [12], suggesting that a careful regimen for treating tumors with EZH2 inhibitors should be considered to maximize the benefits.

Given the complex background of EZH2 in gliomas, we addressed this issue in DMG, using in vivo mouse models, with histone H3 WT (wild type) and *Ezh2* manipulation. In the loss-of-function model, the CRE recombinase was used to create *Ezh2* deletion (exon 14–15, *Ezh2*^f/f^, EZH2 loss-of-function, EZH2 LOF) mice and the EZH2 overexpression was established by breeding the Ntv-a and *Ezh2*^Y641F^ mice [45]. We found that *Ezh2* loss triggers a transcriptional program characterized by an upregulation of the inflammatory interferon gamma response and correlated with a severe disease phenotype. Our *Ezh2* gain-of-function model, on the other hand, reduced tumor incidence and significantly improved survival along with a positive enrichment of the oxidative phosphorylation pathway. Pharmacological inhibition of EZH2 in vitro accelerated cellular proliferation consistent with our *Ezh2* loss-of-function genetic model, whereas in vivo chemical inhibition of EZH2 had limited efficacy on proliferative index or overall survival. Our data suggest a potential tumor suppressive role for EZH2 in DMG that suggests careful investigation of the use of EZH2 inhibitors in the clinic.

## Materials and methods

### Mice

Nestin-Tva (Ntv-a) and Ntv-a; p53^fl/fl^ mice have been described earlier [3, 19]. Ezh2^fl^ mice (B6;129S1-*Ezh2*^*tm2Sho*^/J, JAX stock #022,616; The Jackson Laboratory) have *loxP* sites flanking exons 14–15 that result in deletion of the SET domain (critical for the methyltransferase activity) by *Cre* recombinase [43]. These mice were bred with Ntv-a mice to establish the Ntv-a;*Ezh2*^fl^ genetic background, hereafter referred as EZH2 WT (infected with RCAS-PDGF-B, RCAS-shp53, RCAS-Y viruses). Mice infected with the RCAS CRE virus along with the RCAS-PDGF-B and RCAS-shp53 viruses that resulted in *Ezh2* deletion (Ntv-a;*Ezh2*^f/f^) are referred hereafter as *Ezh2* LOF (*Ezh2* loss-of-function). For the gain-of-function studies, mice with the *Ezh2*^Y641F^ mutation (Dr. N. Sharpless, National Cancer Institute), [45]) were bred with Ntv-a mice to establish the Ntv-a;*Ezh2*^Y641F^ genotype, hereafter referred as EZH2 WT (infected with RCAS-PDGF-B, RCAS-shp53, RCAS-Y viruses). Mice infected with the RCAS CRE virus along with the RCAS-PDGF-B and RCAS-shp53 viruses that resulted in *Ezh2* overexpression (Ntv-a;*Ezh2*^Y641F^) are referred hereafter as EZH2 GOF (*Ezh2* gain-of-function). We also crossed Ntv-a;p53^fl/fl^; with the conditional EZH2^fl/fl^ described above to generate Ntv-a; p53 ^fl/fl^; EZH2 ^fl/fl^. For the EZH2 LOF RNAseq experiments, we infected both Ntv-a;p53^fl/fl^ and Ntv-a; p53 ^fl/fl^; EZH2 ^fl/fl^ with RCAS-PDGFB and RCAS-Cre thus comparing p53 deleted tumors to p53 deleted tumors with EZH2 LOF. Lastly, Ntv-a;p53^fl/fl^; *abcb*1a^−/−^; *abcb*1b^−/−^; *abcg*2^−/−^ (ABC *knockout,* ABC KO*)* mice were bred as described earlier [31]. REDExtract-N-Amp Tissue PCR Kit (Sigma) was used to isolate genomic DNA from tail samples per the manufacturer’s protocol using primers as previously published [3]. All work with mice was done in accordance with the Northwestern University/Lurie Children’s Animal Care and Use Committee and the Guide for the Care and Use of Laboratory Animals (protocol IS00005131). DMGs were established using the replication-competent avian sarcoma-leukosis virus (ASLV) long terminal repeat (LTR) with splice acceptor that enters the cell using the Tumor Virus A (TVA, RCAS-Tv-a system). Avian DF1 fibroblast cells infected with RCAS viruses expressing PDGF-B, CRE and shp53 constructs were injected intracranially as described earlier [30]. For the ABC KO mice, RCAS-PDGF-B, CRE and H3.3K27M virus producing DF1 cells were injected to establish DMG tumors. Injected mice were monitored and weighed every 2 days or more frequently as determined by health criteria and euthanized with CO_2_ when presented with signs of brain tumor (enlarged head, altered gait, weight loss of up to 25%). For the treatment regimen, Tazemetostat (0.5% Na-CMC + 0.1% Tween-80, EPZ-6438, SelleckChem) was administered by the intraperitoneal route at 400 mg/kg b.w for 7 days, prior to disease symptom manifestation.

### Human patient samples

DMG patient samples (n=11) were collected at autopsy under the IRB protocol 2019-3164. EZH2 and Ki-67 slides were stained (Discovery Ultra, Ventana Medical System, Roche Diagnostics) using the antibodies listed in Additional File 1 and tumor areas were quantified using the Halo quantitative image analysis platform (Indica Labs) in the Biorepository and Pathology Core laboratory at the Icahn School of medicine at Mount Sinai.

### In vitro brainstem neurosphere infection with RCAS viruses

Brainstem progenitors were harvested from Ntv-a;*Ezh2*^fl^ postnatal day 3 (P3) pups and processed using papain and ovomucoid to generate a single cell suspension [30]. Cells were seeded in 6-well plates (Corning) with complete media consisting of NeuroCult™ Neural Stem Cell Media (StemCell Technologies) supplemented with 10% NeuroCult™ Proliferation Supplement (StemCell Technologies), 1% Pen–Strep (Invitrogen), 20 ng/mL human basic FGF (Invitrogen), 10 ng/mL human EGF (Invitrogen), and 2 μg/mL heparin (Stem Cell Technologies) and incubated at 37 °C with 5% CO_2_. RCAS viruses were concentrated following the manufacturer’s instructions (Clontech) and added to the cells after 24 h. Neurospheres were harvested after 7 days using ACCUTASE™ cell detachment solution and cells were seeded in 96-well plates. EZH2 inhibitor EPZ011989 was added after 24 h for the next 5 days at the desired concentration (1 µM, twofold step dilution starting at 5 µM) and proliferation was measured using the Cell Proliferation ELISA, BrdU (colorimetric, Roche) kit.

### Immunohistochemistry (IHC) and tumor grading

Tumor tissues were fixed in 10% formalin and embedded in paraffin at the Northwestern University Mouse Histopathology Laboratory. Sections were cut at 5 μm thickness using a Leica RM2235 microtome. Hematoxylin and Eosin (H&E) staining was performed according to established protocols. Tumor grading was performed by a blinded neuropathologist (D.B). Tumors were graded by the WHO grading. IHC analysis was performed using an automated processor (Discovery Ultra, Ventana, Roche Diagnostics). Antibodies used for IHC analysis are listed in Additional File 1. Images were captured on a Zeiss Axio A1 microscope and processed using the Zen 2.3Lite software. Nuclear staining was quantitated on the BioTek Lionheart FX automated microscope using the Gen5 software version 3.08 (BioTek). The quantification of the EZH2 IHC in the EZH2 LOF model was performed using Neurolucida imaging software (MBF Bioscience).

### RNA-sequencing (RNA-seq)

The stranded total RNA-seq was conducted at the Northwestern University NUSeq Core Facility. Briefly, total RNA examples were checked for quality on Agilent Bioanalyzer 2100 and quantitated with Qubit fluorometer. The Illumina TruSeq Stranded Total RNA Library Preparation Kit was used to prepare sequencing libraries. The kit procedure was performed without modifications. This procedure includes rRNA depletion, remaining RNA purification and fragmentation, cDNA synthesis, 3’ end adenylation, Illumina adapter ligation, library PCR amplification and validation. lllumina HiSeq 4000 Sequencer was used to sequence the libraries with the production of single-end, 50 bp reads. The quality of reads, in fastq format, was evaluated using FastQC (Andrews S. (2010). FastQC: quality control tool for high throughput sequence data. Available online at: http://www.bioinformatics.babraham.ac.uk/projects/fastqc). Adapters were trimmed, and reads of poor quality or aligning to rRNA sequences were filtered using Trim Galore (http://www.bioinformatics.babraham.ac.uk/projects/trim_galore/). The cleaned reads were aligned to the mouse genome (mm10) using STAR [14]. Read counts for each gene were calculated using HTSeq-Counts [1] in conjunction with a gene annotation file for mm10 obtained from Ensembl (http://useast.ensembl.org/index.html). A comprehensive QC report was generated using MultiQC [15]. For the LOF EZH2 RNAseq analysis, the number of reads aligning to each Ezh2 or Tp53 exon was quantified from aligned BAM files using DEXSeq [2]. The Emsembl mm10 gtf file was used as input. Exon values were normalized to the total number of aligned reads in the BAM file and are included in Additional file 8: Table S7. DESeq2 analyses were run comparing EZH2 LOF or EZH2 GOF mice to EZH2 WT mice [28]. The cutoff for determining significantly differentially expressed genes was an FDR-adjusted p-value less than 0.05. The pathway analysis was done using Metascape [52]. Gene set enrichment analysis (GSEA) was run using genes ranked according to the Wald statistic with the following parameters: permutations = 1000, enrichment statistic = classic, max size = 500, min size = 20, normalization mode = meandiv. Custom GSEA gene lists were generated from the pedcbioportal by taking the top 100 genes positively co-expressed with IDH1, IDH2, IDH3, PSMB8, PSMB9, and PSMB10 in low-grade glioma samples (n = 259), high-grade glioma samples (n = 133), and DMG samples (n = 97) from the Pediatric Brain Tumor Atlas. GSEA was run using the parameters described above to compare *Ezh2*^*f/f*^or *Ezh2*^Y641F^ mice to control mice based on the custom gene lists.

### Real-time PCR (RT-PCR)

Total RNA was isolated from primary tumors using the RNeasy mini kit (Qiagen) per the manufacturer’s instructions. For semi-quantitative and qRT-PCR validation, cDNA was synthesized from total mRNA using Superscript II and Oligo dT primers (Invitrogen). Primers for validation of *Ezh2* exon 14–15 deletion have been described earlier [34]. All qRT-PCR experiments were performed using triplicate wells and data analyzed on the QuantStudio 6 Flex instrument (Applied Biosystems). Details of all primers are provided in the Additional File 1. Relative expression of target genes was analyzed using the ΔΔC_t_ method (QuantStudio Real-time PCR software v1.3).

### Immunoblot analysis

Snap frozen tumors were lysed in RIPA buffer containing 1 × Protease Inhibitor Cocktail (Sigma Aldrich), 10 mM PMSF, 50 mM NaF, 1 mM NaVO4, and 1 mM DTT and sonicated (QSonica, 5 s, 50% amplitude). Protein estimation was performed using the Pierce™ BCA protein assay kit (ThermoFisher Scientific) and quantified on the Cytation5 plate reader (BioTek). Histone extracts were prepared using the Histone extraction kit following manufacturer’s instructions (Abcam). Nuclear or whole cell lysates were loaded on 4–20% Mini-Protean TGX gels (BioRad) and transferred to Immuno-Blot PVDF membrane (BioRad). Membranes were incubated with primary antibodies at a 1:1000 dilution in Odyssey Blocking Buffer (LI-COR) with 0.2% Tween-20 overnight at 4 °C. Secondary antibodies (1:10,000 for IRDye800CW or 1:20,000 for IRDye680LT) were incubated at room temperature for 1 h. Antibodies and uncropped images of representative blots used for western blot analysis are listed/shown in the Additional File 1.

### Statistical analysis

Statistical analysis was performed using GraphPad Prism software (Version 8.0). All data are represented as the mean with SEM and analyzed using unpaired Student’s *t* test. Sample sizes are indicated in the figure legends. Survival curves were analyzed by log-rank (Mantel–Cox) test. For measuring tumor incidence Fisher’s exact test (two-tailed) was used. Statistical significance was recorded when the *p* value was less than 0.05. Pearson correlation calculations and correlation plot was performed using Prism software.

## Results

### Genetic ablation of Ezh2 aggravates DMG progression that is curtailed by Ezh2 overexpression

**Fig. 1.**
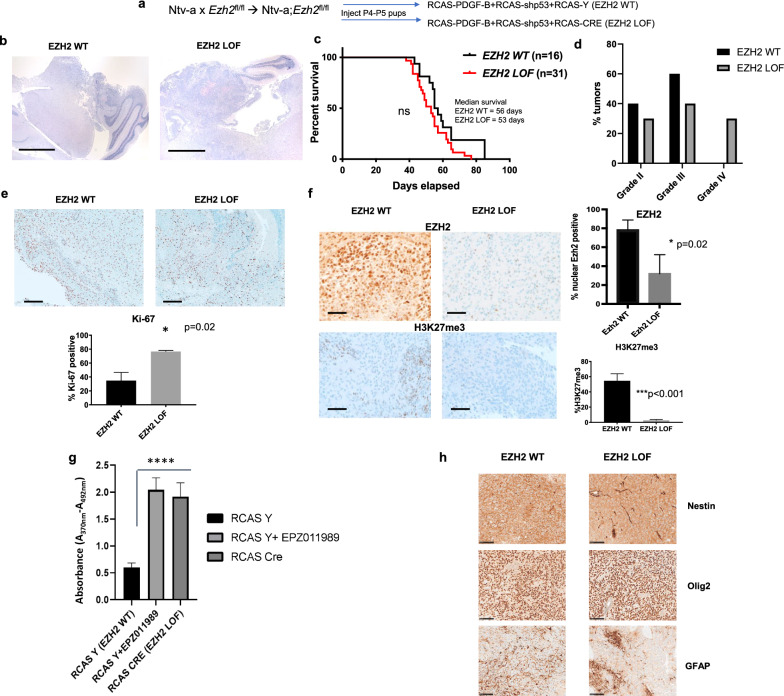
*Ezh2* loss worsens DMG pathogenesis while overexpression delays the disease. DMGs was induced by injecting DF1 cells infected with RCAS-PDGF-B, CRE, shp53-RFP and RCAS-Y/RCAS-CRE viruses in P4 pups from Ntv-a;*Ezh2*^fl/fl^ mice. **a**. Schematic representing establishment of EZH2 loss-of-function DMG mouse model. **b**. Representative H&E staining of tumors from EZH2 WT and EZH2 LOF mice, scale bar = 500 µm. **c.** Kaplan–Meier survival curve of EZH2 WT and EZH2 LOF mice, *p* = 0.0847 by Gehan-Breslow-Wilcoxon test,*ns*, not significant. **d.** Tumor grades determined in both groups are shown. **e.** Representative Ki-67 staining from both groups is shown, scale bar = 100 µm (top) and quantitation of staining, (*p* = 0.02, unpaired *t*-test n = 5 tumors/group, bottom). **f.** Representative EZH2 and H3K27me3 staining from both the groups is shown, scale bar = 100 µm and quantitation of EZH2 (*p* = 0.02, unpaired t-test n = 3 tumors/group) and H3K27me3, (*p* < 0.001, unpaired *t*-test n = 5 tumors/group). **g.** Quantitation of BrdU incorporation by cell proliferation assay after treatment with EPZ011989 (1 µM) in P3 neural precursor cells infected with RCAS-PDGF-B, RCAS-shp53RFP and RCAS-Y/RCAS-CRE viruses, (bottom, *p* < 0.0001, unpaired *t*-test, n = 5 independent experiments). **h.** Representative IHC staining for both groups for Nestin, Olig2 and GFAP (scale bar = 100 µm)

In the *Nestin* promoter-driven model, CRE mediated deletion of* Ezh2* (*Ezh2*^f/f^, RCAS CRE, EZH2 LOF, Fig. 1a) accelerated the development of DMG, including WHO group IV tumors (necrotic, pseudopalisading, active mitotic cells, Fig. 1b and d), as evaluated by a blinded neuropathologist (DB). Mice with *Ezh2* deletion succumbed to the disease earlier with a median survival of 53 days as compared to 56 days in the control *Ezh2*^fl/fl^ RCAS-Y/EZH2 WT group (Fig. 1c, *ns*, not significant, *p* = 0.0847). Although the survival difference did not reach statistical significance, the hazard ratio of EZH2 LOF/EZH2 WT was 1.88, implying that subjects with an *Ezh2* loss of function were almost twice as likely to succumb to the disease earlier as compared to those in the *Ezh2* wild-type (WT) group. Approximately 20% of the tumors in the *Ezh2* deleted cohort were ‘high grade’/Grade IV (Fig. 1d). Proliferative index quantitated by IHC analyses of Ki-67 staining showed a significantly higher percentage of positive cells in EZH2 LOF compared to EZH2 WT (EZH2 LOF = 75% vs. EZH2 WT = 38%, *p* = 0.02, Fig. 1e). *Ezh2* deletion was validated by the loss of EZH2 expression (EZH2 LOF = 31.9% vs. EZH2 WT = 78.3% %, *p* = 0.02) and a near complete loss of H3K27me3 as evaluated by IHC staining (Fig. 1f). In brainstem neural progenitors, *Ezh2* deletion resulted in significantly higher proliferation compared to the EZH2 WT group (RCAS Y), that was mirrored when cells were treated with an EZH2 inhibitor, EPZ011989 (1 µM, Fig. 1g, *p* < 0.0001). Tumors in both groups were positive for Nestin and Olig2, while GFAP expression was sparse (Fig. 1h). Tumor incidence, however, was not different between the groups (Additional file 1: Fig. S1a). Interestingly, we did not register a complete deletion of the *Ezh2* exon 14–15 using the RCAS-CRE system as detected by qualitative PCR, either in formalin-fixed paraffin embedded (FFPE) tumor tissues or in *in-vitro* cultured P3 neuronal precursors (Additional file 1: Fig. S1b, [34]).

**Fig. 2:**
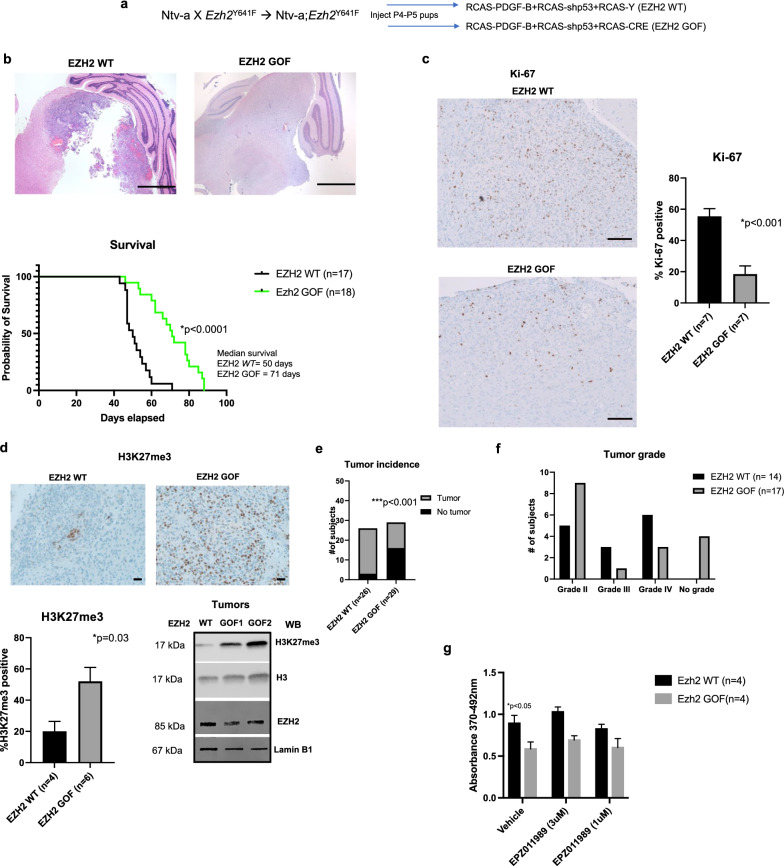
**a** Schematic representation to show establishment of the EZH2 overexpression DMG mouse model. **b.** Representative H&E images from EZH2 WT and EZH2 GOF mice (top), scale bar = 500 µm and (bottom) Kaplan–Meier survival curves of mice from both the groups (*p* < 0.0001, log-rank test). **c.** Representative Ki-67 IHC staining from EZH2 WT and EZH2 GOF tumors and quantitation of staining (*p* < 0.001, unpaired *t*-test n = 3 tumors/group), scale bar = 50 µm **d.** Representative H3K27me3 staining in EZH2 WT and EZH2 GOF tumors, quantitation of H3K27me3 staining (*p* = 0.03, paired *t*-test n = 3 tumors/group) and immunoblot detection of H3K27me3, H3 (loading control) in histone extracts of tumors and EZH2 and Lamin B1 (loading control) from whole cell extracts of tumors from EZH2 WT and EZH2 GOF mice, scale bar 2 µm **e.** Tumor incidence comparison between EZH2 WT and EZH2 GOF mice (*p* < 0.001, Fisher’s exact test). **f.** Tumor grades across the EZH2 WT and EZH2 GOF mice were determined as described. **g.** Cell proliferation BrdU ELISA assay of neuronal precursor cells from Ntv-a;*Ezh2*^Y641F/+^ P3 pups infected with RCAS-PDGF-B, RCAS-shp53-RFP and RCAS-Y/RCAS-CRE viruses and treated with EZH2 inhibitor, EPZ011989 (p < 0.05, unpaired *t*-test, n = 3 independent experiments)

To strengthen our loss-of-function studies, we next investigated the effects of *Ezh2* overexpression on DMG development in *Ezh2*^Y641F^ mice using RCAS viruses as described in “Materials and Methods” (Fig. 2a). Note that the mutant *Ezh2*^Y641F/+^ (referred as *Ezh2*^Y641F^ or EZH2 GOF) is genetically equivalent to clinical cases where the heterozygous mutation is sufficient to induce a disease phenotype. Mice homozygous for the mutation developed hydrocephalus and failed to survive beyond 6 weeks of age. *Ezh2*^Y641F^ /EZH2 GOF overexpression significantly delayed DMG development as compared to the EZH2 WT control group, (Fig. 2b top and bottom, median survival 71 days in RCAS-CRE/EZH2 GOF group compared to 50 days in RCAS-Y/EZH2 WT, *p* < 0.0001, log-rank test). This was reflected in the significantly diminished Ki-67 staining observed in the *Ezh2*^*Y*641F^ /EZH2 GOF cohort (Fig. 2c, *p* < 0.001), along with enhanced H3K27me3 staining and protein expression (Fig. 2d top and bottom, *p* = 0.03). There was a 50% preponderance of subjects in *Ezh2*^Y641F^ /EZH2 GOF cohort for not developing tumors (Fig. 2e, *p* < 0.001, Fisher’s exact test), and many of these tumors represented conditions designated by the pathologist as ‘no grade’ with ‘mild mitotic activity of neuronal cells’ (Fig. 2f). There were significantly more tumors in this group that were lower grade as compared to the control group (EZH2 WT). These results were mirrored in *‘*in vitro*’* assays where overexpression of EZH2 reduced the proliferative capacity of neural precursors (Fig. 2g, *p* < 0.05), although further inhibition by an EZH2 inhibitor (EPZ011989) was not registered. However, RNA-Seq results indicated that only 1 out of 4 tumors expressed the *Ezh2*^Y641F^ allele (Additional file 2: Table S1). The reasons for this are not clear but may be because RNA-seq is not a 100% efficient technique to detect mutations, especially when the gene is expressed at a low level, and the mutant allele fraction is low. Tumors in both groups were positive for Olig2 while GFAP expression was limited/absent as seen in earlier studies (Additional file 1: Fig. S2a). These results support our hypothesis that *Ezh2* likely behaves as a tumor suppressor in DMG.

### Loss of Ezh2 triggers components of the immunoproteasome pathway

**Fig. 3:**
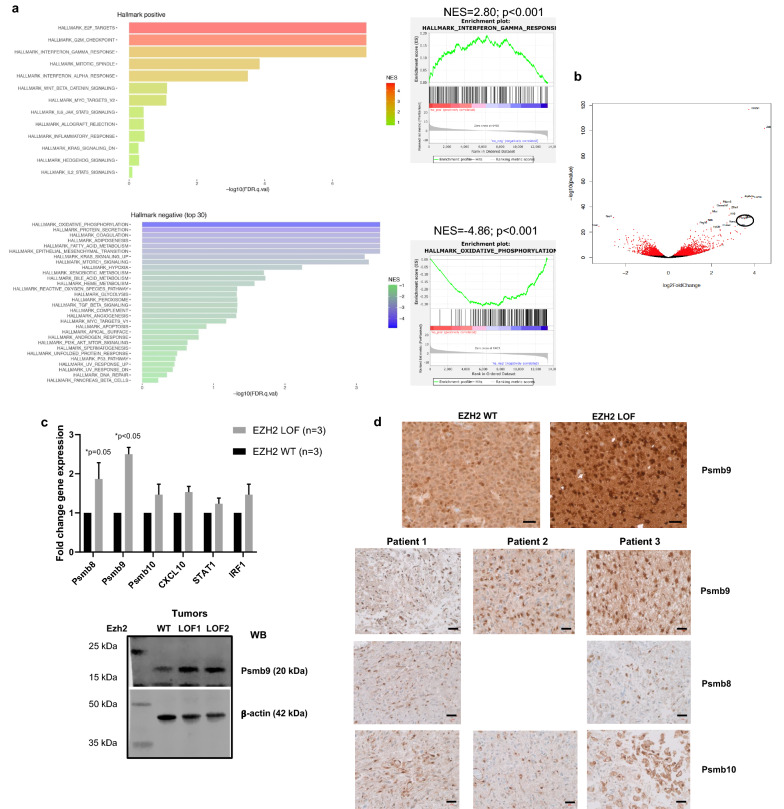
GSEA ranking and validation of targets identified by RNA-sequencing in EZH2 LOF tumors. **a.** Hallmark positive enrichment for ‘E2F targets’, ‘G2M checkpoint’ and ‘interferon gamma response’ (top) and negative enrichment for ‘oxidative phosphorylation’ (bottom) bar plots are shown. **b.** Volcano plot showing the enrichment of genes in the EZH2 LOF tumors, *Psmb9* is highlighted. **c.** Validation of RNA-seq targets by qRT-PCR (top, *p* < 0.05, unpaired *t*-test, n = 3 tumors/group) and western blot (bottom) shows significant expression of immunoproteasome genes (*Psmb9* fold change by qPCR and immunoblot = 2.5). **d.** Representative staining of EZH2 WT and EZH2 LOF tumors for Psmb9 (top) and human DMG samples for Psmb9, 8 and 10 (bottom). Scale bars are 20 µm

Transcriptional profiling by bulk RNA-seq of tumors driven by PDGF-B; p53 loss compared to PDGF-B; p53 loss, and EZH2 loss revealed 3,553 significantly differentially expressed genes. Of these, 1740 were significantly upregulated by EZH2 loss and 1,813 were significantly downregulated (Additional file 3: Table S2). GSEA revealed an over-representation of *‘interferon alpha/gamma pathway genes’,* among *‘E2F targets’* and *‘G2M checkpoint’* in *Ezh2* deleted tumors along with a significant down-regulation of *‘oxidative phosphorylation’* indicating a possible suppression of the mitochondrial metabolic pathway (Fig. 3a, Additional file 6: Table S5). This is consistent with previous reports of heightened aerobic glycolysis and subdued mitochondrial activity in DMG [42].

Among the *‘interferon alpha/gamma response pathway genes*’, genes of the immunoproteasome complex, *Psmb9*, were most notably upregulated (Fig. 3b, highlighted). Validation experiments by qRT-PCR and immunoblot in tumors from mice with *Ezh2* deletion (*Ezh2*^f/f^) confirmed significant overexpression of Psmb9 (LMP2, Fig. 3c top and bottom, *p* < 0.05). IHC staining of control (EZH2 WT) and *Ezh2* deleted (EZH2 LOF) tumors and human DMG autopsy sections confirmed an overall higher proportion of cells positive for Psmb9 (LMP2) in comparison to Psmb8 (LMP7) and Psmb10 (MECL-1, Fig. 3d). These results support a positive contribution of the immunoproteasome system towards DMG development in the event of an *Ezh2* functional loss.

### Idh1 induction in Ezh2 overexpression model shows a protective effect in curtailing DMG

**Fig. 4:**
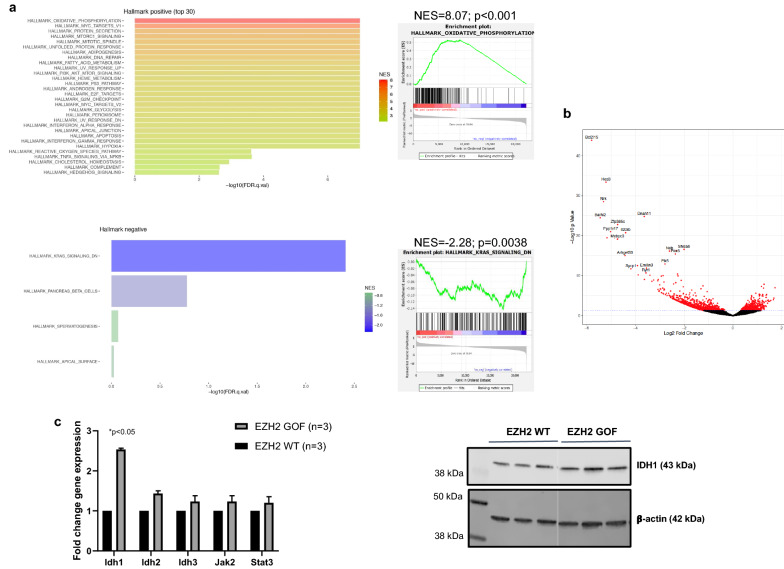
GSEA ranking and validation of targets identified by RNA-sequencing in EZH2 GOF tumors. **a.** Hallmark positive enrichment for ‘oxidative phosphorylation’ and negative enrichment for ‘KRAS signaling’ bar plots and scores are shown. **b.** Volcano plot showing the enrichment of genes in the EZH2 GOF tumors. **c.** Validation of RNA-seq targets by qRT-PCR (top, *p* < 0.05, unpaired *t*-test, n = 3 tumors/group) and cropped images of western blot (bottom) show significant expression of *Idh1* (qPCR fold change = 2.5)

Overexpression of the *Ezh2*^Y641F^ mutant allele significantly prolonged survival of mice injected with DF1 cells producing RCAS-PDGF-B, RCAS-CRE, and RCAS-shp53 viruses. We performed RNA-seq of EZH2 WT and EZH2 GOF (n = 3 and n = 4 respectively) tumor samples. We identified 294 significantly differentially expressed genes with 227 genes being downregulated and 67 genes being upregulated in EZH2 GOF tumors relative to EZH2 WT (Additional file 4: Table S3). GSEA analysis of the bulk RNA-Seq of these tumors revealed *‘oxidative phosphorylation’* as the most significantly upregulated pathway (Fig. 4a, Additional files 5: Table S4, Additional files 7: Table S6). Among the genes from the oxidative phosphorylation pathway, *Idh1* was modestly upregulated as verified by qRT-PCR and immunoblotting (Fig. 4b, *p* < 0.05). These results suggest a protective role of *Idh1* in *Ezh2* overexpression induced delayed DMG development. Interestingly we did not observe any significant changes to the immunoproteasome genes in the EZH2 overexpression model.

### In vivo pharmacological inhibition of Ezh2 in an ABC KO mouse DMG model

**Fig. 5:**
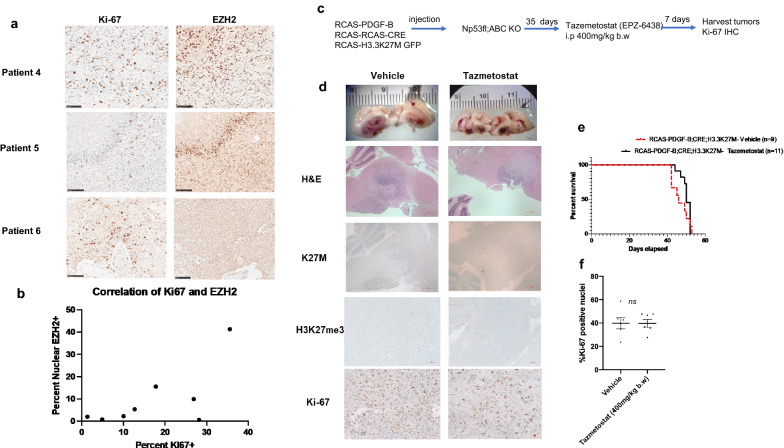
EZH2 expression in DMG and pharmacological inhibition in an in vivo DIPG model. **a.** Ki-67 and EZH2 expression are shown in representative DMG patient samples (scale bar = 100 µm). **b.** Correlation plot demonstrating a positive correlation between EZH2 and Ki67 in human DMG patient samples with r = 0.6774, n = 8, *p* value 0.0649 by a 2 tailed test **c.** Tazemetostat (EPZ-6438) treatment protocol for Np53fl; ABC KO mice injected with RCAS-PDGF-B, CRE and H3.3K27M virus producing DF1 cells to establish DMG is shown. **d.** Representative H&E and IHC staining of tumors for K27M, H3K27me3, and Ki-67 from vehicle and Tazemetostat treated mice are shown. Scale bar for H&E and K27M IHC is 500 µm, 100 µm for H3K27me3 IHC, and 20 µm for KI-67 IHC **e.** Kaplan–Meier survival curve comparison between vehicle and Tazemetostat treated mice is depicted. Median survival for the vehicle group was 46 days and 50 days for the Tazemetostat arm with a p-value of 0.25 by the log-rank test. **f.** Quantitation of Ki-67 score in both groups is shown, n.s *not significant*

Recent studies have suggested that EZH2 is a potential target in DMG [32, 39], substantiated by the recent FDA approval of Tazemetostat (Tazverik™, Epizyme) for epithelioid sarcoma and follicular lymphoma. We interrogated the clinical significance of EZH2 expression and whether targeting EZH2 is a viable therapeutic option in DMG. Immunohistochemical staining of DMG patient samples showed a positive correlation between the expression of Ki-67 and EZH2 r = 0.6774, n = 8, however the relationship was not significant with a *p* = 0.0649 by two-tailed test (Fig. 5a, b). To investigate the effect of EZH2 inhibition in a DMG in vivo tumor model, we established mutant H3.3K27M tumors in the ABC KO mice, and treated intraperitoneal (i.p) with Tazemetostat (EPZ-6438, SelleckChem, 400 mg/kg b.w.) for 7 days to enable drug penetration through the blood–brain barrier [31, 50]. We chose to use the ABC KO mouse as it has been reported by others that the brain penetration of EPZ-6438 is limited by abcb1 [50]. Treatment with EPZ-6438 did not alter Ki-67 staining when compared to vehicle treated tumors (Fig. 5d and f) but modestly increased the median survival from 46 days in vehicle treated mice to 50 days in the drug treated cohort (*p* = 0.09 by Gehan-Breslow-Wilcoxon test, hazard ratio (vehicle/Taz) = 2.0, (Taz/vehicle) = 0.49, Fig. 5e). In summary, short-term treatment of our murine DMG model with tazemetostat did not significantly impact the proliferation rate of the tumor cells nor significantly prolonged survival. The short duration of treatment and the aggressive model are potential reasons for the lack of significant efficacy.

## Discussion

EZH2 is suggested to have a pro-tumorigenic role in gliomas (both pediatric and adult). Specifically, in DMG where 80% of cases express the mutant H3K27M histone, focal gains of the H3K27me3 mark on critical tumor suppressors such as *Cdkn2a* [11] among others have been implied as an indirect consequence of residual EZH2 activity [32]. Pharmacological inhibition of this residual activity by several EZH2 inhibitors and its consequence on arresting DMG cellular proliferation lends support to this notion. However, there are caveats to these studies. For example, in the study by Mohammad et al., loss of *Trp53* nullifies effects of any inhibition by EZH2 inhibitors [32]. This is clinically significant in determining which subset of DMG patients receive such treatment as approximately 75% DMG cases present with a loss of a functional Trp53 [22]. On the other hand, a case of H3WT DMG with a Y646H EZH2 has been reported as well [37]. Additionally, as there are no known mutations of the PRC2 genes in high grade gliomas, the mechanism of the effect of EZH2 remains context dependent [24]. Given this complex background we attempted to discern the role of EZH2 in DMG using loss- and gain-of-function genetic mouse models.

Loss of *Ezh2* clearly enhanced tumor grade and proliferative index, albeit it did not significantly alter the tumor latency when compared to the EZH2 WT group. A similar observation was made by Neff et al., who showed that primary inactivation of *Ezh2* in leukemic cells bestowed a growth advantage but did not lend any survival benefit to primary recipients [34]. It was only after serial selection of *Ezh2-null* cells and transplantation into secondary recipients that *Ezh2* was found to be completely deleted and a survival advantage was witnessed. In our RCAS Tv-a system, using *Cre* recombinase resulted in a partial deletion of the *Ezh2* exon 14–15 (verified by qualitative PCR and RNA-Seq). We acknowledge that this is a major limitation of the study. Nevertheless, even with the possibility that only a subset of cells had the *Ezh2* allele deleted, there was a near complete loss of H3K27me3 staining in the tumors generated from this cohort. Given this heterogeneity in the system, we established DMG tumors in an *Ezh2* gain-of-function model using the mutant *Ezh2*^Y641F^ (murine equivalent of the human Y646H mutant allele) mice. Overexpression of *Ezh2* significantly delayed DMG development that was reflected in the significantly decreased tumor incidence and reduced proliferation rate of these tumors. Most of these tumors could not be categorized in the ‘classical low grade’ and, therefore, ascertained as ‘no tumor’ even though the mice were symptomatic with tumors, with an exaggerated H3K27me3 staining and protein presence that confirmed overexpression of the mutant *Ezh2*^Y641F^ allele. Overall, our observations suggest a tumor suppressive role of *Ezh2* in DMG, even though there are isolated clinical cases of DMG which have reported a similar gain-of-function *Ezh2* mutation [37]. A highly context-dependent function of *Ezh2* has come to light in a recent work by Basheer et al., where *Ezh2* deletion prior to retroviral transduction with oncogenic *MLL-AF9* accelerated disease development and shortened survival in mouse models of acute myeloid leukemia (AML).[4]. However, this effect was reversed when oncogenic transformation was initiated prior to *Ezh2* deletion. These results hint at temporal control by EZH2 in determining an oncogenic outcome during development. Clinically, AML patients with a loss-of-function *EZH2* mutation do not show a promising survival advantage when compared to their *EZH2* WT counterparts. Hence using EZH2 inhibitors against a carefully selected group of patients stratified on the basis of their *EZH2* mutation status is highly recommended [44].

Our *in-vitro* experiments of EZH2 chemical inhibition in P3 brainstem neural progenitors infected with RCAS viruses mirrored the observations of accelerated cell growth seen with genetic deletion of *Ezh2.* Recent studies have also reported that EZH2 inhibition in murine neural stem cells or human DMG cell lines by pharmacological inhibitors works best at nanomolar concentrations whereas higher doses induce proliferation of cells [18]. Genetic modeling of DMG is valuable in evaluating responses to potential drugs such as EZH2 inhibitors as genetic models present the endogenous disease with altered murine genetics rather than relying on orthotopic models that depend on human DMG cell lines [41]. With this in mind, we established brainstem gliomas in Np53fl; ABC KO mice to overcome the blood–brain barrier related efflux of EZH2 inhibitor, EPZ-6438. In a short-term treatment protocol, we did not observe any difference in the proliferative index of tumors between the vehicle and drug treated groups, and there was no significant difference in survival between the two cohorts. It is unclear if the lack of in vivo efficacy is due to limited drug penetration, or the aggressive nature of this model. We did note a positive correlation between expression of EZH2 and Ki67 in patient samples although the correlation was not significant like due to the small patient cohort consistent with a similar trend observed by others [21]. We could not confirm active drug penetration in the brain tissues of the treated mice, and this remains to be verified. There have been studies reporting no cytotoxic response using EZH2 inhibitor Tazemetostat in cell lines [46] that align with our findings; however, Zhang et al., showed that using JQ-1 (BET inhibitor) in combination with Tazmetostat, there was a significant decrease in cell proliferation and a survival benefit in animals bearing PDGF-B/H3.3K27M tumors. However, there is no information on the status of p53 in these tumors and the neural stem cells used in this study were from the dorsal forebrain [51]. Thus, these studies appear to be inconclusive on whether EZH2 inhibitors would be efficacious in patients with DMGs.

To gain clarity on the gene expression programs perturbed by alterations in *Ezh2*, we performed bulk RNA-Seq and discovered that suppression of the oxidative phosphorylation pathway in *Ezh2* loss-of-function tumors (NES = − 4.85; FDR *p* < 0.001) and significant positive enrichment of the oxidative phosphorylation pathway (NES = 8.07; FDR *p* < 0.001) in the *Ezh2* gain-of-function cohort as the most robust pathway impacted by EZH2 genetic perturbation. In addition, a robust interferon gamma response program was upregulated in both the EZH2 LOF and GOF models. Among the overrepresented genes in the interferon pathway were those from the immunoproteasome complex namely Psmb8 (LMP7), Psmb9 (LMP2), and Psmb10 (MECL-1). These proteasome genes are critical to the MHC Class-I related antigen processing pathway, located in the vicinity of the MHC class II genomic region and are highly regulated by interferon-γ treatment especially in non-immune cells [33]. Overexpression of immunoproteasomes is well documented in multiple myeloma, prostate and some forms of breast cancer, and is a poor prognostic marker; hence, there is an interest in targeting this pathway in the clinic [49]. In glioblastomas, Psmb8 (LMP7) inhibition decreased tumor angiogenesis and was designated as an independent unfavorable prognostic marker [8]. There are no previous reports of immunoproteasome expression in pediatric high-grade gliomas particularly in DMG. Our results show high expression of Psmb9 in *Ezh2* deleted murine DMG tumors as well as in patient samples. However, a systematic study is needed to analyze if there is any correlation between expression of EZH2 and genes of the immunoproteasome pathway. In melanomas, the presence of these proteins indicates a favorable response to checkpoint blockade as they mark increased infiltration of patient-matched tumor infiltrating lymphocytes [20]. Currently there is a Phase I safety and efficacy study recruiting pediatric patients with DMG for a combination treatment of marizomib (brain penetrating proteasome inhibitor) and panobinostat (NCT04341311).

Our gain-of-function RNA-seq analysis identified an upregulated oxidative phosphorylation pathway, comprising of members of the isocitrate dehydrogenase family, the *Idh1/2/3* genes. Wild-type isocitrate dehydrogenases (IDH1 and IDH2) catalyze the oxidative carboxylation of isocitrate to α-ketoglutarate. By acquiring a neomorphic enzymatic activity that results in a gain-of-function mutation, IDH1R132H, implicated in low grade gliomas, converts isocitrate to 2-hydroxy ketoglutarate (2-HG), an oncometabolite [6, 40], However, the more documented IDH^R132H^ is also predicted to act as a tumor suppressor in glioma by creating a synthetic lethal vulnerability to DNA damage inducing radiosensitization [35]. The fact that wild-type IDH1 is causally related to clinical cases of low-grade gliomas lends support to our own findings of its upregulation in the *Ezh2* gain-of-function ‘tumors’, presenting similar features. An earlier study has shown that knockdown of wild-type IDH1 triggers a set of tumor suppressor genes, including NDUFS1 (murine *Ndufa9),* which when mutated is implicated in low survival in certain cancers [7], and there is a report of high expression of WT-IDH1 in H3K27M tumors [9]. But we found a positive correlation between *Idh1* and *Ndufa9* in our *Ezh2* gain-of-function tumors.

In summary, our observations using *Ezh2* loss- and gain-of-function DMG mouse models suggest a ‘tumor suppressor’ role of EZH2 in DMG. These are further substantiated by the discovery of the transcriptional programs ensued upon genetic perturbation of *Ezh2*, namely elevated interferon gamma response in the loss-of-function and gain-of-function cohort and an augmented oxidative phosphorylation/mitochondrial metabolism signature in the gain-of-function genetic model. Further studies are warranted to understand the role of these pathways in DMG, including similar studies in other DMG model systems such as the recently described in utero model or using this same model but with PDGFA ligand overexpression instead of PDGFB [36]. Genome wide H3K27me3 distribution studies are planned in the future to better characterize how oxidative phosphorylation is regulated by EZH2, and to identify which genes are directly regulated by EZH2. Cautious support is recommended for translation of EZH2 inhibitors for the benefit of DMG patients.

## Conclusions

This study was aimed to understand the role of EZH2 in H3WT DMG. Though a few recent investigations suggest that EZH2 could play a pro-tumorigenic role in gliomas, specifically in DMG, the exact contribution of EZH2 in this disease is lacking partly due to the absence of appropriate in vivo mouse models. Using an *Ezh2* loss-and-gain-of-function approach in the RCAS-Tv/a system, this study shows that EZH2 could function as a potential tumor suppressor. Transcriptomic analysis revealed candidate gene signatures that support an aggravated disease phenotype visualized in *Ezh2*-deleted subjects, while the gain-of-function slowed disease progression. Therefore, a careful selection of patients is recommended when considering treatment using *Ezh2* inhibitors. Although further functional validation is warranted, this is the first study to elucidate the complex role of EZH2 in DMG.

## Supplementary Information

**Additional file 1.** Supplemental Figures & Tables.

**Additional file 2.** Y641F read counts of EZH2 GOF and control EZH2 WT RNAseq samples..

**Additional file 3.** List of significantly differentially expressed genes comparing EZH2 LOF vs. EZH2 WT in the context of PDGFB, and p53 loss.

**Additional file 4.** List of significantly differentially expressed genes comparing EZH2 GOF vs. EZH2 WT in the context of PDGFB and p53 shRNA.

**Additional file 5.** Ranking of significantly differentially enriched gene-sets by GSEA enriched in opposite directions in EZH2 GOF vs. EZH2 WT comparison and EZH2 LOF vs. EZH2 WT comparison.

**Additional file 6.** GSEA analysis of EZH2 LOF vs. EZH2 WT in the context of PDGFB, and p53 loss.

**Additional file 7.** GSEA analysis of EZH2 GOF vs. EZH2 WT in the context of PDGFB and p53 shRNA.

**Additional file 8.** Exon quantification of EZH2 and TP53 of RNAseq samples in the EZH2 LOF vs. EZH2 WT in the context of PDGFB and p53 loss.

## Data Availability

All data generated or analyzed in this manuscript are included in the article and its supplementary material. All request for data or supporting material may be sent to the corresponding author. Datasets generated from this publication have been deposited in the GEO (GSE188450).

## Abbreviations

Ezh2: Enhancer of zeste homolog 2
PRC2: Polycomb repressive complex 2
pHGG: Pediatric high-grade glioma
DMG: Diffuse midline glioma
Ntv-a: Nestin- Tva
RCAS: Replication competent avian sarcoma-leucosis
LTR: Long terminal repeat
TVA: Tumor virus A
PDGFB: Platelet-derived growth factor B
IHC: Immunohistochemistry
RNA-seq: RNA-sequencing
GSEA: Gene set enrichment analysis
i.p: Intraperitoneal
µM: Micromolar
NES: Normalized enrichment score
FDR: False discovery rate

## References

[1] Anders S, Pyl PT, Huber W (2015). HTSeq–a Python framework to work with high-throughput sequencing data. Bioinformatics.

[2] Anders S, Reyes A, Huber W (2012). Detecting differential usage of exons from RNA-seq data. Genome Res.

[3] Barton KL, Misuraca K, Cordero F, Dobrikova E, Min HD, Gromeier M, Kirsch DG, Becher OJ (2013). PD-0332991, a CDK4/6 inhibitor, significantly prolongs survival in a genetically engineered mouse model of brainstem glioma. PLoS ONE.

[4] Basheer F, Giotopoulos G, Meduri E, Yun H, Mazan M, Sasca D, Gallipoli P, Marando L, Gozdecka M, Asby R (2019). Contrasting requirements during disease evolution identify EZH2 as a therapeutic target in AML. J Exp Med.

[5] Bender S, Tang Y, Lindroth AM, Hovestadt V, Jones DT, Kool M, Zapatka M, Northcott PA, Sturm D, Wang W (2013). Reduced H3K27me3 and DNA hypomethylation are major drivers of gene expression in K27M mutant pediatric high-grade gliomas. Cancer Cell.

[6] Bhavya B, Anand CR, Madhusoodanan UK, Rajalakshmi P, Krishnakumar K, Easwer HV, Deepti AN, Gopala S (2020). To be Wild or Mutant: Role of Isocitrate Dehydrogenase 1 (IDH1) and 2-Hydroxy Glutarate (2-HG) in Gliomagenesis and Treatment Outcome in Glioma. Cell Mol Neurobiol.

[7] Calvert AE, Chalastanis A, Wu Y, Hurley LA, Kouri FM, Bi Y, Kachman M, May JL, Bartom E, Hua Y (2017). Cancer-associated IDH1 promotes growth and resistance to targeted therapies in the absence of mutation. Cell Rep.

[8] Chang HH, Cheng YC, Tsai WC, Chen Y (2020). PSMB8 inhibition decreases tumor angiogenesis in glioblastoma through vascular endothelial growth factor A reduction. Cancer Sci.

[9] Chung C, Sweha SR, Pratt D, Tamrazi B, Panwalkar P, Banda A, Bayliss J, Hawes D, Yang F, Lee HJ (2020). Integrated metabolic and epigenomic reprograming by H3K27M mutations in diffuse intrinsic pontine gliomas. Cancer Cell.

[10] Comet I, Riising EM, Leblanc B, Helin K (2016). Maintaining cell identity: PRC2-mediated regulation of transcription and cancer. Nat Rev Cancer.

[11] Cordero FJ, Huang Z, Grenier C, He X, Hu G, McLendon RE, Murphy SK, Hashizume R, Becher OJ (2017). Histone H3.3K27M represses p16 to accelerate gliomagenesis in a murine model of DIPG. Mol Cancer Res.

[12] de Vries NA, Hulsman D, Akhtar W, de Jong J, Miles DC, Blom M, van Tellingen O, Jonkers J, van Lohuizen M (2015). Prolonged Ezh2 depletion in glioblastoma causes a robust switch in cell fate resulting in tumor progression. Cell Rep.

[13] Di Croce L, Helin K (2013). Transcriptional regulation by Polycomb group proteins. Nat Struct Mol Biol.

[14] Dobin A, Davis CA, Schlesinger F, Drenkow J, Zaleski C, Jha S, Batut P, Chaisson M, Gingeras TR (2013). STAR: ultrafast universal RNA-seq aligner. Bioinformatics.

[15] Ewels P, Magnusson M, Lundin S, Kaller M (2016). MultiQC: summarize analysis results for multiple tools and samples in a single report. Bioinformatics.

[16] Feinberg AP, Koldobskiy MA, Gondor A (2016). Epigenetic modulators, modifiers and mediators in cancer aetiology and progression. Nat Rev Genet.

[17] Fontebasso AM, Schwartzentruber J, Khuong-Quang DA, Liu XY, Sturm D, Korshunov A, Jones DT, Witt H, Kool M, Albrecht S (2013). Mutations in SETD2 and genes affecting histone H3K36 methylation target hemispheric high-grade gliomas. Acta Neuropathol.

[18] Haag D, Mack N, Benites Goncalves da Silva P, Statz B, Clark J, Tanabe K, Sharma T, Jager N, Jones DTW, Kawauchi D (2021). H3.3-K27M drives neural stem cell-specific gliomagenesis in a human iPSC-derived model. Cancer Cell.

[19] Holland EC, Hively WP, DePinho RA, Varmus HE (1998). A constitutively active epidermal growth factor receptor cooperates with disruption of G1 cell-cycle arrest pathways to induce glioma-like lesions in mice. Genes Dev.

[20] Kalaora S, Lee JS, Barnea E, Levy R, Greenberg P, Alon M, Yagel G, Bar Eli G, Oren R, Peri A (2020). Immunoproteasome expression is associated with better prognosis and response to checkpoint therapies in melanoma. Nat Commun.

[21] Karlowee V, Amatya VJ, Takayasu T, Takano M, Yonezawa U, Takeshima Y, Sugiyama K, Kurisu K, Yamasaki F (2019). Immunostaining of increased expression of enhancer of zeste homolog 2 (EZH2) in diffuse midline glioma H3K27M-mutant patients with poor survival. Pathobiology.

[22] Khuong-Quang DA, Buczkowicz P, Rakopoulos P, Liu XY, Fontebasso AM, Bouffet E, Bartels U, Albrecht S, Schwartzentruber J, Letourneau L (2012). K27M mutation in histone H3.3 defines clinically and biologically distinct subgroups of pediatric diffuse intrinsic pontine gliomas. Acta Neuropathol.

[23] Kim E, Kim M, Woo DH, Shin Y, Shin J, Chang N, Oh YT, Kim H, Rheey J, Nakano I (2013). Phosphorylation of EZH2 activates STAT3 signaling via STAT3 methylation and promotes tumorigenicity of glioblastoma stem-like cells. Cancer Cell.

[24] Kim KH, Roberts CW (2016). Targeting EZH2 in cancer. Nat Med.

[25] Knutson SK, Kawano S, Minoshima Y, Warholic NM, Huang KC, Xiao Y, Kadowaki T, Uesugi M, Kuznetsov G, Kumar N (2014). Selective inhibition of EZH2 by EPZ-6438 leads to potent antitumor activity in EZH2-mutant non-Hodgkin lymphoma. Mol Cancer Ther.

[26] Knutson SK, Warholic NM, Wigle TJ, Klaus CR, Allain CJ, Raimondi A, Porter Scott M, Chesworth R, Moyer MP, Copeland RA (2013). Durable tumor regression in genetically altered malignant rhabdoid tumors by inhibition of methyltransferase EZH2. Proc Natl Acad Sci U S A.

[27] Lewis PW, Muller MM, Koletsky MS, Cordero F, Lin S, Banaszynski LA, Garcia BA, Muir TW, Becher OJ, Allis CD (2013). Inhibition of PRC2 activity by a gain-of-function H3 mutation found in pediatric glioblastoma. Science.

[28] Love MI, Huber W, Anders S (2014). Moderated estimation of fold change and dispersion for RNA-seq data with DESeq2. Genome Biol.

[29] Mackay A, Burford A, Carvalho D, Izquierdo E, Fazal-Salom J, Taylor KR, Bjerke L, Clarke M, Vinci M, Nandhabalan M (2017). Integrated molecular meta-analysis of 1,000 Pediatric high-grade and diffuse intrinsic pontine glioma. Cancer Cell.

[30] Misuraca KL, Barton KL, Chung A, Diaz AK, Conway SJ, Corcoran DL, Baker SJ, Becher OJ (2014). Pax3 expression enhances PDGF-B-induced brainstem gliomagenesis and characterizes a subset of brainstem glioma. Acta Neuropathol Commun.

[31] Mittapalli RK, Chung AH, Parrish KE, Crabtree D, Halvorson KG, Hu G, Elmquist WF, Becher OJ (2016). ABCG2 and ABCB1 limit the efficacy of dasatinib in a PDGF-B-driven brainstem glioma model. Mol Cancer Ther.

[32] Mohammad F, Weissmann S, Leblanc B, Pandey DP, Hojfeldt JW, Comet I, Zheng C, Johansen JV, Rapin N, Porse BT (2017). EZH2 is a potential therapeutic target for H3K27M-mutant pediatric gliomas. Nat Med.

[33] Murata S, Takahama Y, Kasahara M, Tanaka K (2018). The immunoproteasome and thymoproteasome: functions, evolution and human disease. Nat Immunol.

[34] Neff T, Sinha AU, Kluk MJ, Zhu N, Khattab MH, Stein L, Xie H, Orkin SH, Armstrong SA (2012). Polycomb repressive complex 2 is required for MLL-AF9 leukemia. Proc Natl Acad Sci U S A.

[35] Nunez FJ, Mendez FM, Kadiyala P, Alghamri MS, Savelieff MG, Garcia-Fabiani MB, Haase S, Koschmann C, Calinescu AA, Kamran N (2019). IDH1-R132H acts as a tumor suppressor in glioma via epigenetic up-regulation of the DNA damage response. Sci Transl Med.

[36] Patel SK, Hartley RM, Wei X, Furnish R, Escobar-Riquelme F, Bear H, Choi K, Fuller C, Phoenix TN (2020). Generation of diffuse intrinsic pontine glioma mouse models by brainstem-targeted in utero electroporation. Neuro Oncol.

[37] Pfaff E, El Damaty A, Balasubramanian GP, Blattner-Johnson M, Worst BC, Stark S, Witt H, Pajtler KW, van Tilburg CM, Witt R (2019). Brainstem biopsy in pediatric diffuse intrinsic pontine glioma in the era of precision medicine: the INFORM study experience. Eur J Cancer.

[38] Phillips RE, Soshnev AA, Allis CD (2020). Epigenomic reprogramming as a driver of malignant glioma. Cancer Cell.

[39] Piunti A, Hashizume R, Morgan MA, Bartom ET, Horbinski CM, Marshall SA, Rendleman EJ, Ma Q, Takahashi YH, Woodfin AR (2017). Therapeutic targeting of polycomb and BET bromodomain proteins in diffuse intrinsic pontine gliomas. Nat Med.

[40] Reitman ZJ, Parsons DW, Yan H (2010). IDH1 and IDH2: not your typical oncogenes. Cancer Cell.

[41] Sasaki T, Katagi H, Goldman S, Becher OJ, Hashizume R (2020). Convection-enhanced delivery of enhancer of zeste homolog-2 (EZH2) inhibitor for the treatment of diffuse intrinsic pontine glioma. Neurosurgery.

[42] Shen H, Yu M, Tsoli M, Chang C, Joshi S, Liu J, Ryall S, Chornenkyy Y, Siddaway R, Hawkins C (2020). Targeting reduced mitochondrial DNA quantity as a therapeutic approach in pediatric high-grade gliomas. Neuro Oncol.

[43] Shen X, Liu Y, Hsu YJ, Fujiwara Y, Kim J, Mao X, Yuan GC, Orkin SH (2008). EZH1 mediates methylation on histone H3 lysine 27 and complements EZH2 in maintaining stem cell identity and executing pluripotency. Mol Cell.

[44] Skoda RC, Schwaller J (2019). Dual roles of EZH2 in acute myeloid leukemia. J Exp Med.

[45] Souroullas GP, Jeck WR, Parker JS, Simon JM, Liu JY, Paulk J, Xiong J, Clark KS, Fedoriw Y, Qi J (2016). An oncogenic Ezh2 mutation induces tumors through global redistribution of histone 3 lysine 27 trimethylation. Nat Med.

[46] Wiese M, Schill F, Sturm D, Pfister S, Hulleman E, Johnsen SA, Kramm CM (2016). No Significant Cytotoxic Effect of the EZH2 Inhibitor Tazemetostat (EPZ-6438) on Pediatric Glioma Cells with Wildtype Histone 3 or Mutated Histone 3.3. Klin Padiatr.

[47] Wu G, Broniscer A, McEachron TA, Lu C, Paugh BS, Becksfort J, Qu C, Ding L, Huether R, Parker M (2012). Somatic histone H3 alterations in pediatric diffuse intrinsic pontine gliomas and non-brainstem glioblastomas. Nat Genet.

[48] Xu K, Wu ZJ, Groner AC, He HH, Cai C, Lis RT, Wu X, Stack EC, Loda M, Liu T (2012). EZH2 oncogenic activity in castration-resistant prostate cancer cells is Polycomb-independent. Science.

[49] Zerfas BL, Maresh ME, Trader DJ (2020). The Immunoproteasome: an emerging target in cancer and autoimmune and neurological disorders. J Med Chem.

[50] Zhang P, de Gooijer MC, Buil LC, Beijnen JH, Li G, van Tellingen O (2015). ABCB1 and ABCG2 restrict the brain penetration of a panel of novel EZH2-Inhibitors. Int J Cancer.

[51] Zhang Y, Dong W, Zhu J, Wang L, Wu X, Shan H (2017). Combination of EZH2 inhibitor and BET inhibitor for treatment of diffuse intrinsic pontine glioma. Cell Biosci.

[52] Zhou Y, Zhou B, Pache L, Chang M, Khodabakhshi AH, Tanaseichuk O, Benner C, Chanda SK (2019). Metascape provides a biologist-oriented resource for the analysis of systems-level datasets. Nat Commun.

