# Supplementary material for: A tumor suppressor role for EZH2 in diffuse midline glioma pathogenesis
Source: Acta Neuropathol Commun. 2022 Apr 8;10:47. doi: 10.1186/s40478-022-01336-5 (PMC8994223; doi:10.1186/s40478-022-01336-5)
Supplement: Supplementary file 1 — Additional file 1. Supplemental Figures & Tables. [file 40478_2022_1336_MOESM1_ESM.docx]

**Additional File 1**

Supplemental Figure 1a: Tumor incidence comparison between Ntv-a; Ezh2^fl/fl^ (RCAS-Y) and Ntv-a; Ezh2^f/f^ (RCAS-CRE)

Supplemental Figure 1b: Ezh2 Exon 14-15 deletion PCR of FFPE samples and p3 neurospheres

Supplemental Figure 2a: Representative IHC for Olig2 and GFAP of EZH2 WT and EZH2 GOF tumor samples

Supplementary Table 1: Antibodies for western blot and IHC

Supplementary Table 2: Primers for Real Time PCR validation

Figure S1a

**Tumor incidence**


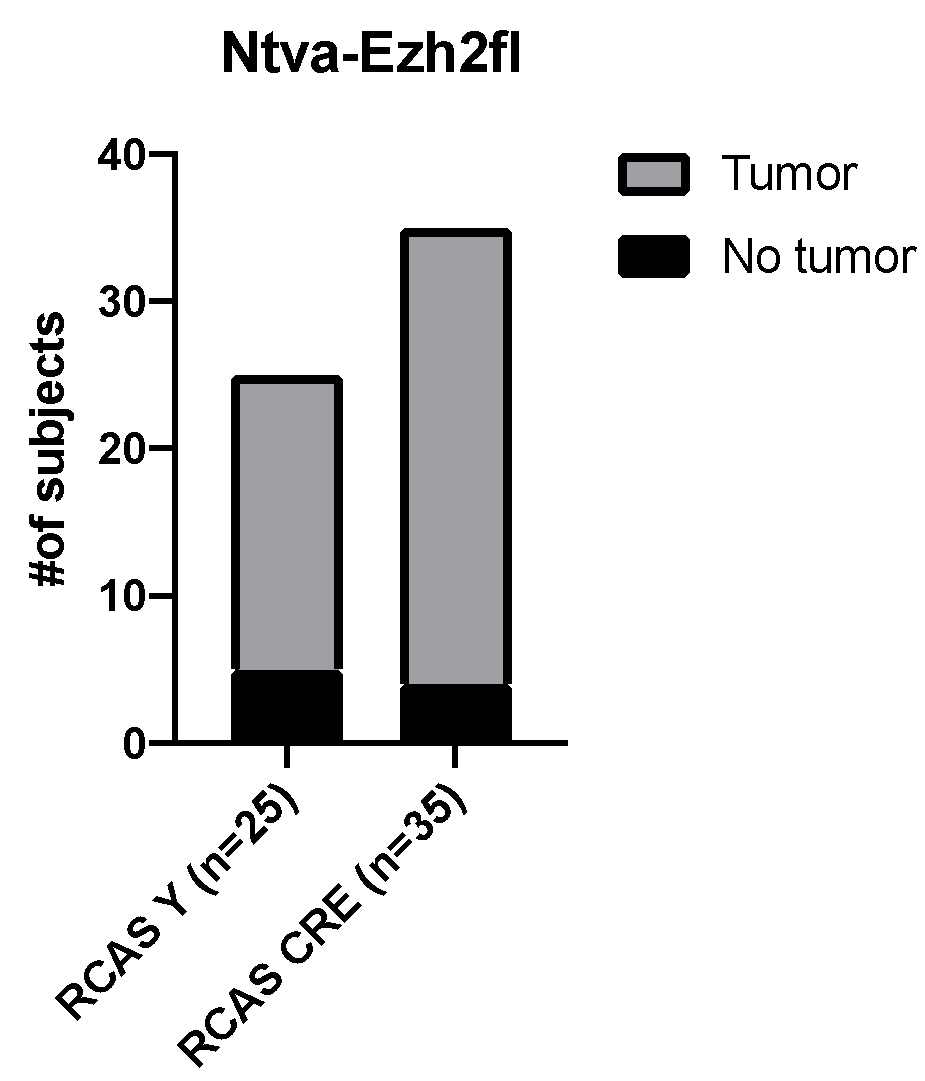


Figure S1b

**Ezh2 exon 14-15 deletion PCR**


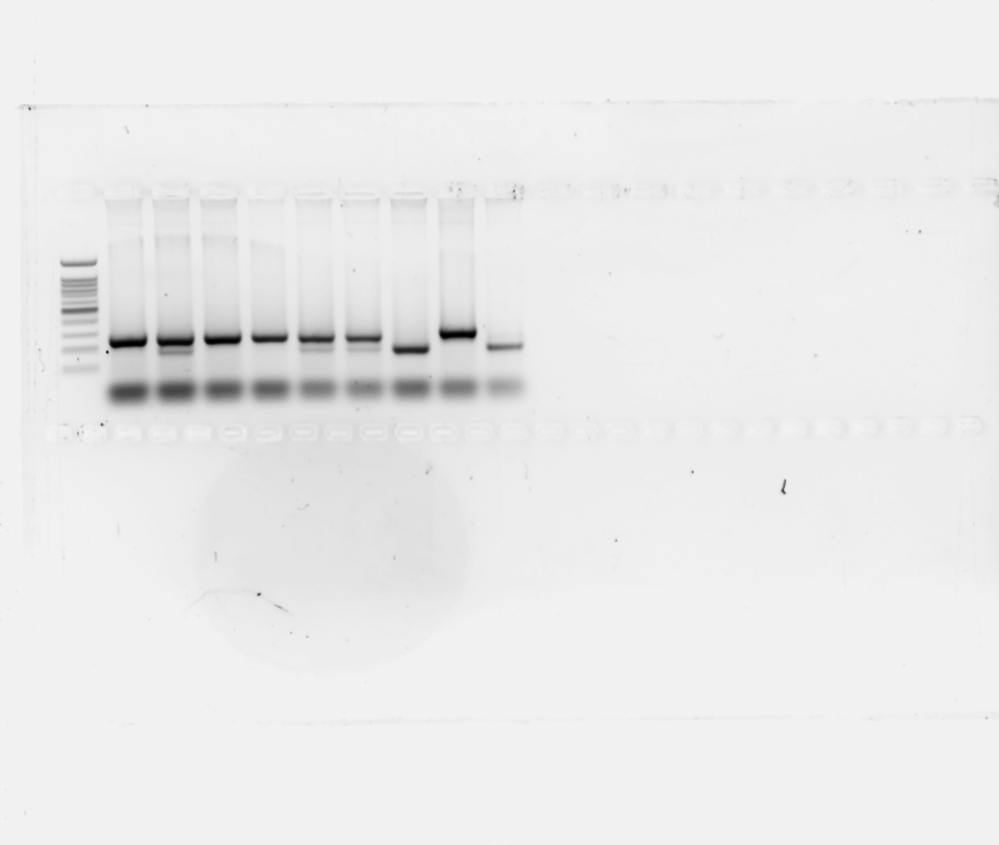


**FFPE**

300

200

**Ezh2^f/f^**


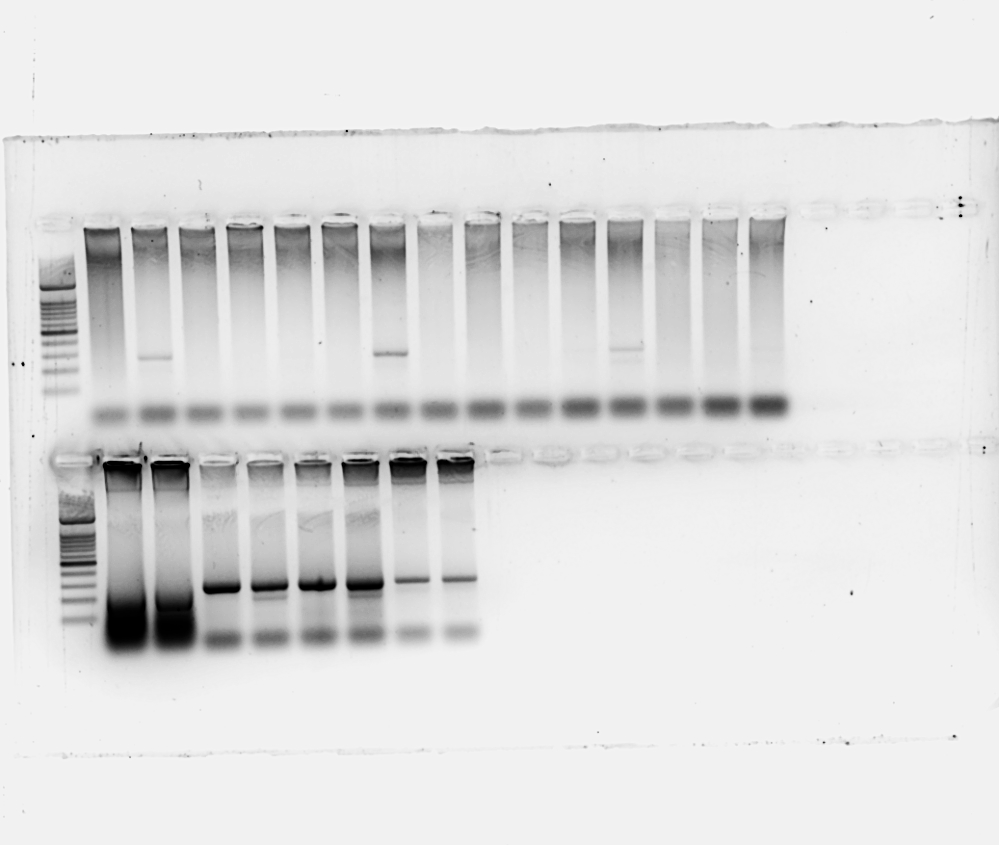

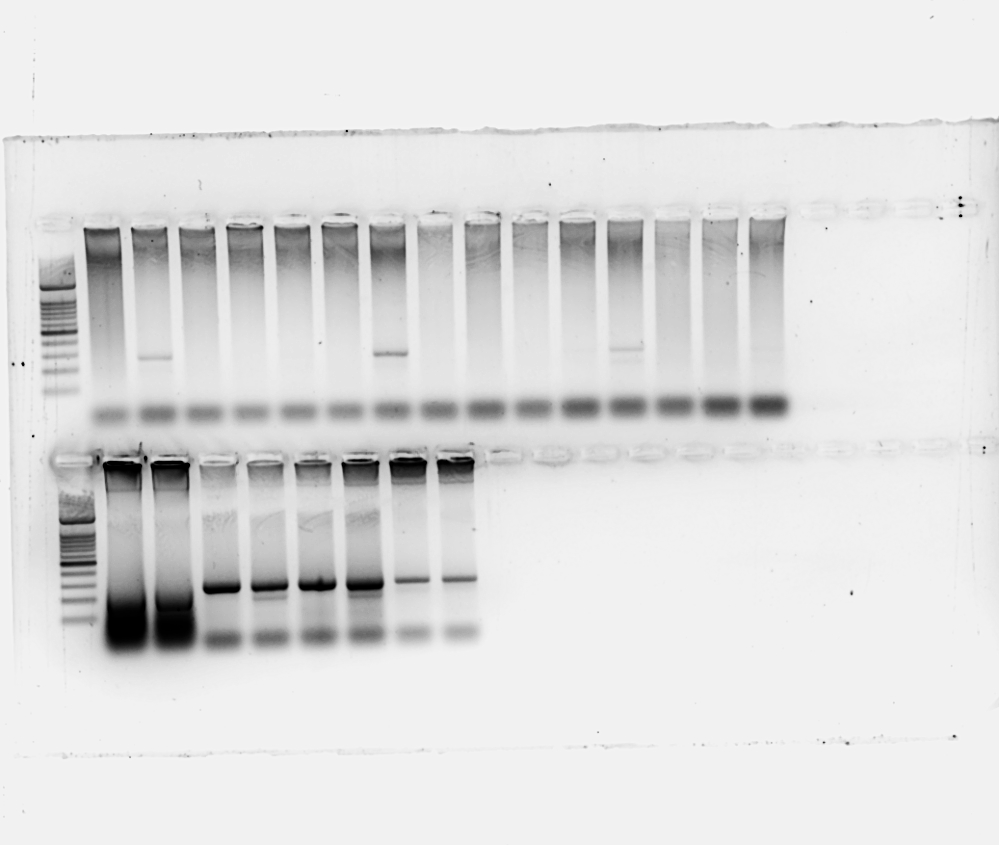

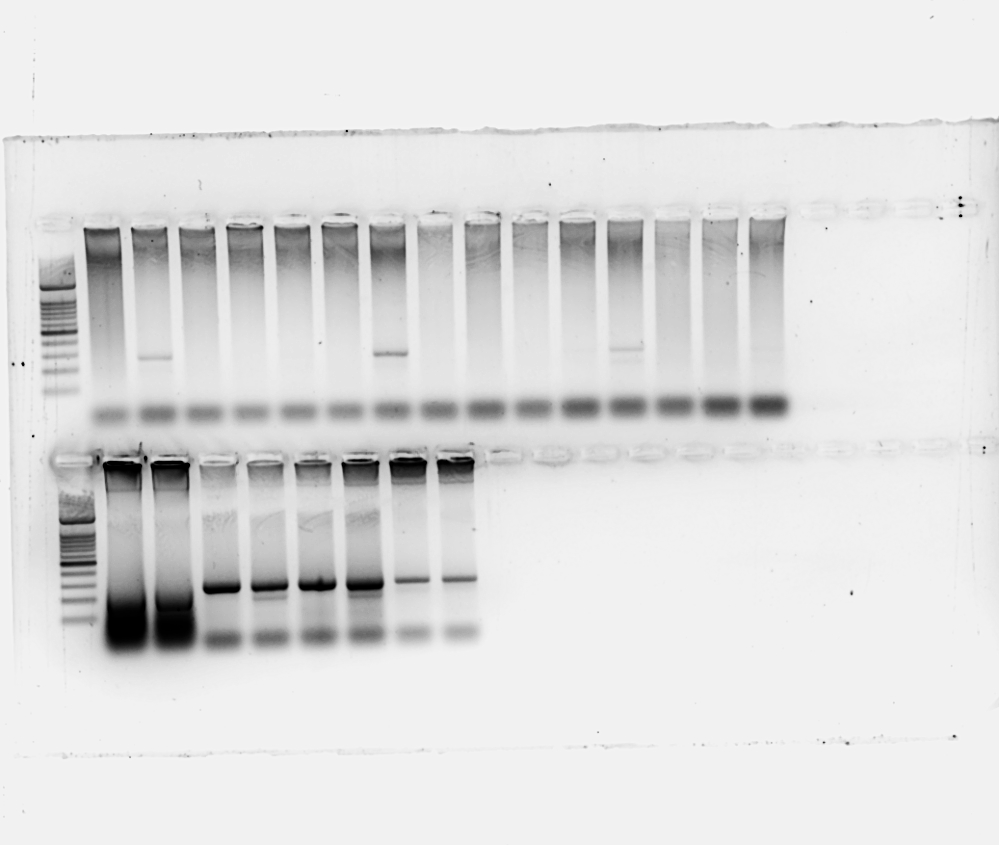


300

200

**Ezh2^fl/fl^**

**P3 Neurospheres**

**Ezh2^fl/fl^**

**Ezh2^f/f^**

Figure S1a. Tumor incidence comparison between Ntv-a;*Ezh2*^fl/fl^ (RCAS-Y) and Ntv-a;*Ezh2*^fl/fl^ (RCAS-CRE). S1b. Ezh2 exon 14-15 deletion PCR[1] in Formalin Fixed Paraffin Embedded (FFPE) sections and in P3 cultured neurosphere cells showing the incomplete deletion of exon-14-15.

Figure S2a

**Ezh2 WT**                                    **Ezh2 GOF**


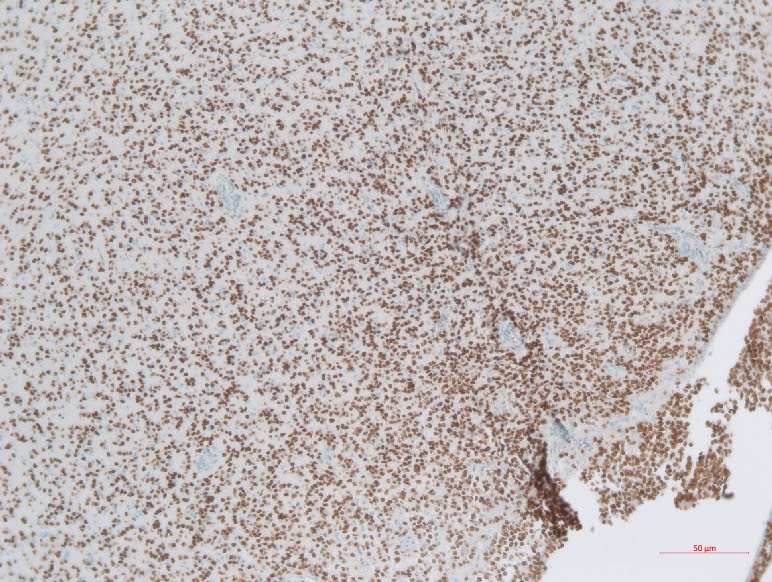

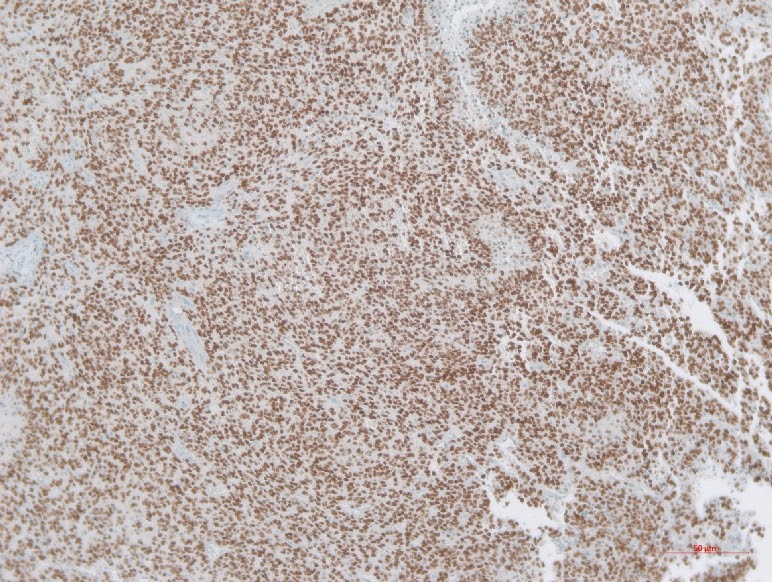
**Olig-2**


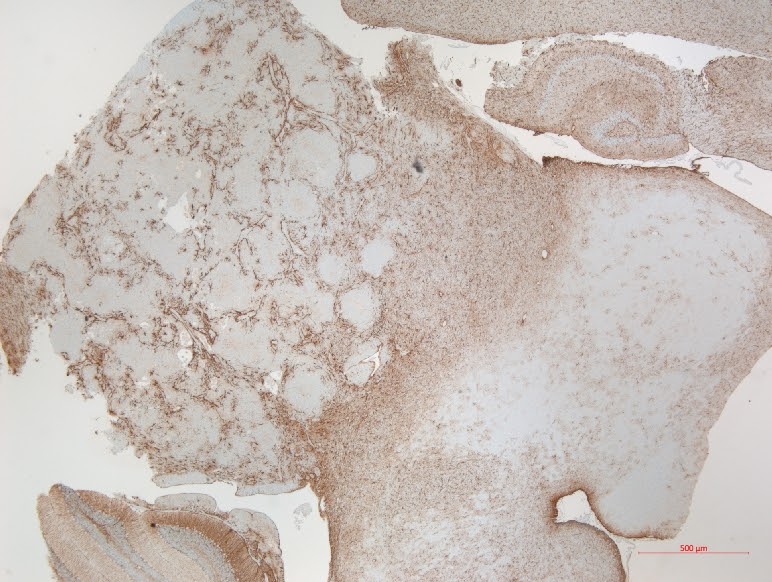

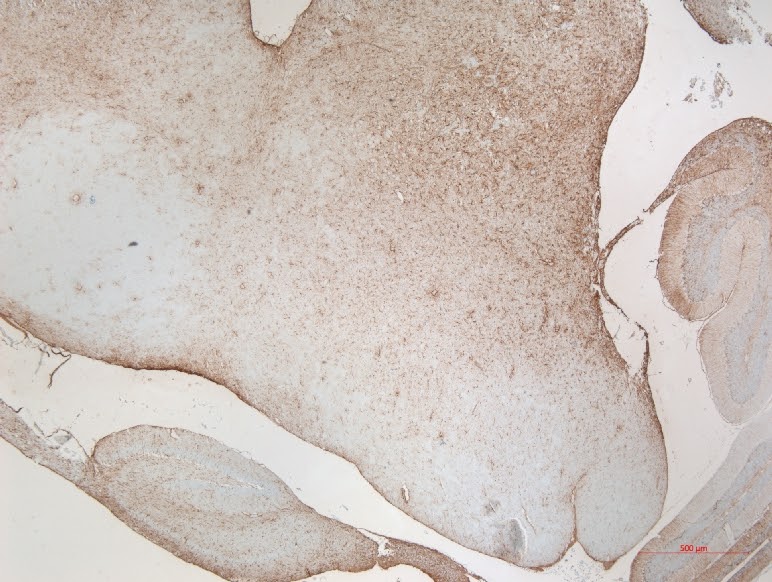
**GFAP**

Figure S2a. Representative IHC images depicting Olig2 and GFAP staining in EZH2 WT and Ezh2 GOF tumor samples. Scale bars are 50µm

**Supplementary Table 1 Antibodies for western blot and IHC**

| **Antibody** | **Company** |
| --- | --- |
| Anti-Tri-Methyl-Histone H3 (Lys27) | Cell Signaling Technology (#9733) |
| Anti-histone H3 | Abcam (ab1791) |
| Anti-KMT6 / EZH2 | Abcam (ab191080) |
| Anti-Lamin B1 antibody nuclear envelope marker | Abcam (ab16048) |
| Anti-Proteasome 20S LMP2 | Abcam (ab242061) |
| Anti-Proteasome 20S LMP7 | Abcam (ab 180606) |
| Anti-Psmb10/MECL1 | Abcam (ab183506) |
| Anti-IDH1 | Abcam (ab172964) |
| Anti-Beta actin | Cell Signaling Technology (#3700) |
| Anti-Ki-67 | Abcam (Ab16667) |
| Anti-Histone H3 (mutated K27M) | Abcam (ab190631) |
| Anti-GFAP | Dako (Z 0334) |
| Anti-Olig2 | Millipore (AB9610) |
| Anti-Nestin | BD Pharmigen (#556309) |

**Supplementary Table 2 Primers for real time PCR validation**

**Ezh2 loss of function**

**Ezh2 Gain of function**

**References**

1 Neff T, Sinha AU, Kluk MJ, Zhu N, Khattab MH, Stein L, Xie H, Orkin SH, Armstrong SA (2012) Polycomb repressive complex 2 is required for MLL-AF9 leukemia. Proc Natl Acad Sci U S A 109: 5028-5033 Doi 10.1073/pnas.1202258109
